# Methamphetamine-induced short-term increase and long-term decrease in spatial working memory affects protein Kinase M zeta (PKMζ), dopamine, and glutamate receptors

**DOI:** 10.3389/fnbeh.2014.00438

**Published:** 2014-12-18

**Authors:** Stephen H. Braren, Damian Drapala, Ingrid K. Tulloch, Peter A. Serrano

**Affiliations:** ^1^Department of Psychology, Hunter College, City University of New YorkNew York, NY, USA; ^2^Department of Psychology, Stevenson UniversityBaltimore, MD, USA; ^3^Department of Psychology, The Graduate Center, City University of New YorkNew York, NY, USA

**Keywords:** methamphetamine, working memory, protein kinase M zeta, dopamine, glutamate receptors, dorsal striatum, hippocampus, radial arm maze

## Abstract

Methamphetamine (MA) is a toxic, addictive drug shown to modulate learning and memory, yet the neural mechanisms are not fully understood. We investigated the effects of 2 weekly injections of MA (30 mg/kg) on working memory using the radial 8-arm maze (RAM) across 5 weeks in adolescent-age mice. MA-treated mice show a significant improvement in working memory performance 1 week following the first MA injection compared to saline-injected controls. Following 5 weeks of MA abstinence mice were re-trained on a reference and working memory version of the RAM to assess cognitive flexibility. MA-treated mice show significantly more working memory errors without effects on reference memory performance. The hippocampus and dorsal striatum were assessed for expression of glutamate receptors subunits, GluA2 and GluN2B; dopamine markers, dopamine 1 receptor (D1), dopamine transporter (DAT) and tyrosine hydroxylase (TH); and memory markers, protein kinase M zeta (PKMζ) and protein kinase C zeta (PKCζ). Within the hippocampus, PKMζ and GluA2 are both significantly reduced after MA supporting the poor memory performance. Additionally, a significant increase in GluN2B and decrease in D1 identifies dysregulated synaptic function. In the striatum, MA treatment increased cytosolic DAT and TH levels associated with dopamine hyperfunction. MA treatment significantly reduced GluN2B while increasing both PKMζ and PKCζ within the striatum. We discuss the potential role of PKMζ/PKCζ in modulating dopamine and glutamate receptors after MA treatment. These results identify potential underlying mechanisms for working memory deficits induced by MA.

## Introduction

Methamphetamine (MA) is a highly addictive drug of abuse that is prevalent among young adults (NIDA, 2012; Talbert, 2014). Clinical studies have identified various cognitive deficits after chronic MA exposure even when followed by years of abstinence (Nordahl et al., 2003; Monterosso et al., 2005; Simon et al., 2010; and Morgan et al., 2012) producing deficits in attention, episodic memory, information processing, and impulse control. MA also produces memory deficits (Simon et al., 2002; Hoffman et al., 2006; Gonzalez et al., 2007) concomitant with reducing hippocampal volume (Orikabe et al., 2011). More surprising is that clinical studies have also identified cognitive-enhancing effects from low doses of MA resulting in enhanced learning and memory performance involving visuospatial perception and response speed after limited and low dose stimulant exposure (Johnson et al., 2000; Silber et al., 2006; Mahoney et al., 2010; Marrone et al., 2010; Hart et al., 2011; Kirkpatrick et al., 2011).

Rodent studies have also found enhancing, short-term effects on cognition from low doses of MA (Moenk and Matuszewich, 2012), an effect specific to adolescent but not adult rats. Low doses of MA exposure during adolescence were found to produce short-term improvements in spatial acquisition but with deficits in spatial short-term working memory performance (McFadden and Matuszewich, 2007). Conversely, exposing rats postnatally over several days impairs spatial reference memory (Vorhees et al., 2000; Williams et al., 2002), but not working memory in adulthood (Williams et al., 2003). These studies indicate that various MA doses can selectively impair reference and working memory, but these effects are dependent on when the drug is delivered and when the behavioral assessments are conducted.

Various MA treatment paradigms are used in rodents to examine the acute and chronic effects on the brain (see reviews Cadet and Krasnova, 2009; Hart et al., 2011). Early signs of neurotoxic damage after MA treatments show selective damage to dopaminergic terminals within the dorsal striatum (Ricaurte et al., 1982, 1984; O'Callaghan and Miller, 1994; Pereira et al., 2002, 2006) and hippocampus (Nash and Yamamoto, 1992; Rocher and Gardier, 2001). Concomitant with dopaminergic terminal damage is a decrease in TH (Sonsalla et al., 1996; Fumagalli et al., 1998; Wallace et al., 1999; Armstrong and Noguchi, 2004; Cadet et al., 2011; North et al., 2013) and DAT levels (Hastrup et al., 2003; Baucum et al., 2004). Correlating these neurochemical effects of MA exposure to cognitive function has identified differences between bolus, binge, and escalating doses of MA exposure (Tulloch et al., 2011b). Several studies show that multiple doses of MA reduced dopamine levels within the striatum but did not result in cognitive impairment (Bisagno et al., 2002; Marshall et al., 2007; Belcher et al., 2008; North et al., 2013) while single day regimens produced cognitive deficits (Friedman et al., 1998; Chapman et al., 2001; Belcher et al., 2005; Marshall et al., 2007; Belcher et al., 2008). These reports suggest that multiple dosages across days may provide some neuroprotection and/or delay the long-term damage (Segal et al., 2003; O'Neil et al., 2006).

We focus our experiments on identifying the progressive effects of MA exposure using weekly spatial working memory assessments to characterize short- and long-term consequences of MA bolus dosages on cognitive function. Our behavioral results show that adolescent mice treated with a bolus dose of MA demonstrate cognitive enhancing effects on a spatial working memory test 1 week after treatment. In the subsequent weeks, these mice were further tested for a spatial cognitive flexibility task in which MA-exposed mice show significantly more working memory errors but not reference memory errors compared to controls. Following all the behavioral assessments we focus our molecular analyses on protein expression patterns within the hippocampus and striatum across three distinct categories that are affected by MA exposure: (1) dopamine receptor 1 (D1), dopamine transporter (DAT) and the precursor to dopamine, tyrosine hydroxylase (TH); (2) glutamate receptors: L-Alpha-amino-3-hydroxy-5-methylisoxazole-4-propionate (AMPA) GluN2B subunit and N-methyl-D-aspartate (NMDA) GluA2 subunit; (3) atypical protein kinase C zeta (PKCζ) and protein kinase M zeta (PKMζ). We focus on these molecular markers since MA selectively damages DA terminals and is known to produce excitotoxic effects involving both AMPA and NMDA receptors (Bowers et al., 2010; Kalivas and Volkow, 2011). PKMζ is an atypical kinase that is important for spatial learning and long-term memory (Serrano et al., 2008; Sebastian et al., 2013b), and increases expression concomitant with improved memory (Sebastian et al., 2013a). Our results identify that 6 weeks after MA abstinence, there are significant protein effects within the hippocampus and striatum, which identify dysregulated expression of dopamine, glutamate, and PKMζ. These data could identify the long-term damage associated with limited MA exposure across multiple brain regions.

## Methods

### Subjects

Male C57BL/6 mice from Taconic Farms (Germantown, NY) were purchased at 7 weeks of age. Subjects were randomly assigned to 2 treatment conditions: MA (*n* = 4) and Saline (*n* = 4). We have used similar sample sizes to evaluate behavioral performance and protein expression as previously reported (Tulloch et al., 2011a; Sebastian et al., 2013a,b). Mice were housed at the Hunter College animal facility for 1 week prior to beginning any behavioral assessments with food and water *ad libitum* prior to behavioral shaping. Mice were housed individually and kept on a 12/12 h light/dark cycle. All housing conditions conform to the Hunter College guidelines outlined by the Institutional Animal Care and Use Committee (IACUC).

### Radial 8-arm maze shaping

The radial 8-arm maze (RAM) was used to assess both working memory (experiment 1), and reference and working memory (experiment 2). The RAM consists of a center platform (15.24 cm diameter) with 8 equivalently sized arms radiating outward. Each arm was 38 cm in length, 6.35 cm wide with a submerged food cup (2.0 cm diameter) at the end of the arm. Maypo (Homestat Farm, Dublin, OH), a sweetened oatmeal, was mixed in water to make a wet mash that was used as a food reward (0.02 g portions), as previously described for rats (Serrano et al., 2008; Sebastian et al., 2013c). Prior to working memory assessments, all animals were shaped on the RAM. Mice were food restricted to 85% of free feeding weight before being placed on the RAM for 10 min to acclimate to the maze and room cues. One hour later, all mice were given a second trial with sweetened oatmeal in the food cups. After 3 days of shaping (2 trials per day), mice were eating the food rewards and finding all 8 baits within a 15 min maximum latency.

### Working memory assessment

Baseline working memory assessment (WMA) occurred over 6 days in which individual mice were tested every other day (3 trials/day) with a 1 h home cage period between trials. Each trial started with all food cups baited. Prior to beginning each trial, mice were confined for 30 s to the center platform with a plastic cylinder. The sequence of arms entered to retrieve the food rewards was recorded. To prevent a non-hippocampal strategy, mice were allowed to collect baits from up to 3 sequential arms before the experimenter interrupted the chaining strategy. Errors were recorded as re-entries into arms where the food reward had been collected. Maximum latency was set at 15 min. After collecting baseline data on working memory assessment, all mice were injected with either MA (30 mg/kg) or saline, delivered intraperitoneally (IP). Weekly working memory assessments were conducted on all mice for 5 weeks following MA treatment. These weekly assessments required that mice only be food restricted the day before testing. On the remaining days all mice were given food chow *ad libitum*.

### Reference and working memory assessment/cognitive flexibility

After 5 weeks of weekly working memory assessments, all mice were then trained on a reference and working memory (RWMA) version of the RAM (Serrano et al., 2008; Sebastian et al., 2013a). This paradigm had 4 baited and 4 unbaited arms in a pattern that was specific to each animal that remained constant throughout the experiment. Mice were given 6 consecutive trials per day for 10 days (60 trials total). Between trials mice were confined to the center platform while the arms were re-baited and the maze cleaned. The sequence of arm entries was recorded. A reference memory error reflected an entry into an arm that was never baited, while a working memory errors reflected re-entries into an arm where the bait had already been collected. Mice were only allowed to enter up to 3 sequential arms to prevent the non-hippocampal, chaining strategy. This version of the RAM required mice to relearn room cues associated with the baited and unbaited arm sequence. The training room and room cues were identical to that used for the WMA. One hour after their 60th trial, brains were microdissected, snap frozen and stored at −80°C.

### Methamphetamine treatment

All mice received a 200 μl injection of either saline or 30 mg/kg (+)—methamphetamine hydrochloride (Sigma Aldrich) delivered IP. Injections of MA or saline took place twice, delivered 1 week apart.

### Tissue fractions

Tissues from hippocampus and dorsal striatum were prepared into cytosolic and synaptic fractions as previously reported (Sebastian et al., 2013a). Briefly, tissues were thawed from frozen and homogenized in a TEE (Tris 50 mM; EDTA 1 mM; EGTA 1 mM) buffer containing a SigmaFast, protease inhibitor cocktail (Sigma Aldrich) diluted to contain AEBSF (2 mM), Phosphoramidon (1 μM), Bestatin (130 μM), E-64 (14 μM), Leupeptin (1 μM), Aprotinin (0.2 μM), and Pepstatin A (10 μM). Tissues were homogenized in 200 μl of the TEE-homogenization buffer using 20 pumps with a motorized pestle (Sacktor et al., 1993). Homogenates were transferred to Eppendorf tubes and centrifuged at 3000 g (5 min at 4°C), to remove the nuclear pellet. The resulting supernatant was centrifuged at 100,000 g for 30 min. After ultracentrifugation, the supernatant was collected and stored as the cytosolic fraction. The remaining pellet was resuspended in 100 μl of homogenizing TEE buffer containing 0.001% Triton X-100, incubated on ice for 1 h and then centrifuged at 100,000 g for 1 h at 4°C. The resulting pellet was resuspended in 100 μl of TEE buffer and stored as the synaptic fraction (Noguès et al., 1994). The Pierce bicinchoninic acid assay (BCA) (Thermo Scientific, Rockford, IL) was used to determine protein concentration for each sample. Samples were reduced with 4× Laemmli sample buffer equivalent to 25% of the total volume of the sample and then boiled and stored frozen at −80°C (Sacktor et al., 1993).

### Immunoblots

Samples (25 μg) were loaded onto a Tris/Gly 8% gel to resolve GAPDH (37 kDa), GluA2 (100 kDa), D1 (48 kDa), and GluN2B (166 kDa), or a 4–20% gradient gel to resolve GAPDH (37 kDa), PKMζ (55 kDa)/PKCζ (70 kDa), TH (58 kDa), and DAT (50 kDa). Gels were transferred to nitrocellulose membranes and were then incubated in blocking solution containing 4% bovine serum albumin (BSA) in Tris Buffered Saline with Tween-20 (TBST; 0.1% Tween-20 in TBS) for 1 h at room temperature. Samples were incubated with the following primary antibodies overnight: GluN2B (1:1000; AbCam, Cambridge, MA), D1 (1:500; AbCam, Cambridge, MA) and with the following primary antibodies for 3 h at room temperature: PKMζ/PCKζ (1:5000; Santa Cruz Biotechnology, Santa Cruz, CA); TH (1:2000; EMD Millipore, Billerica, MA); DAT (1:1000, Santa Cruz Biotechnology; Santa Cruz, CA); GluA2 (1:1000; EMD Millipore, Billerica, MA); and GAPDH: (1:2000, EMD Millipore; Billerica, MA). Blots were rinsed and probed with alkaline-phosphatase coupled secondary antibody and developed with BCIP/NBT substrate (KPL, Gaithersburg, MD). Membranes were scanned for quantification with NIH Image J (Rasband, 2014). Refer to Supplementary Figure 1 for representative immunoblots for target proteins with corresponding molecular weight markers.

### Statistics

For behavioral analyses, a repeated measure, Two-Way ANOVA was used (Prism GraphPad 5.0b Statistical Package, La Jolla, California). Post-hoc analyses used a Bonferroni-corrected *t*-test. Western Blot analyses between MA and control treatments used independent samples *t-*tests.

## Results

**Figure 1:**
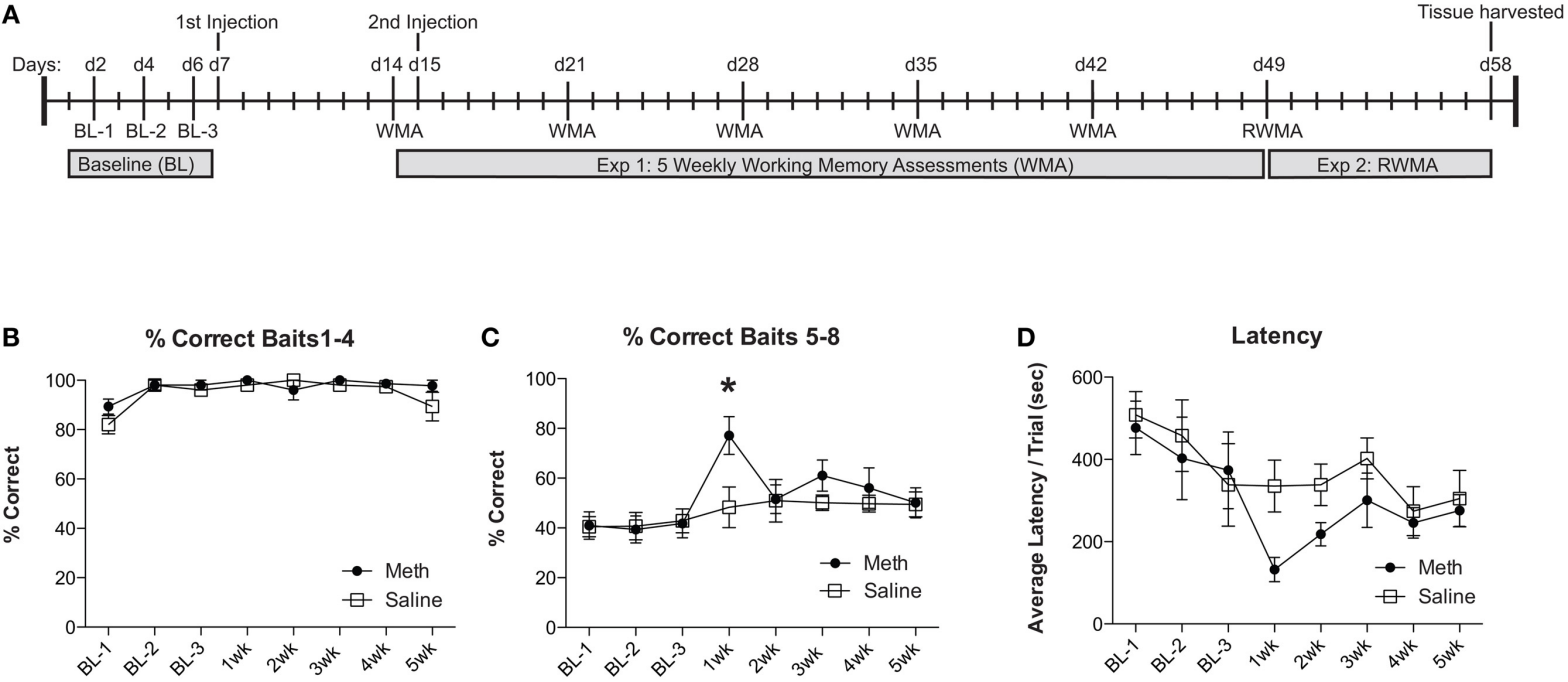
**MA enhances working memory performance acutely. (A)** Timeline for experiments 1 and 2 are illustrated across days. **(B)** Percent correct scores for retrieving the baits 1–4 were not significantly different between conditions and did not significantly change over time. **(C)** Weekly working memory assessments (WMA) show no significant changes in saline controls over the 5 weeks. MA injected mice show significant working memory improvements 1 week after the first injection (^*^p < 0.05). Over the remaining weeks, the elevated performance in MA treated mice returns to baseline. **(D)** Latency to complete the task shows a significant effect of time but no significant effects of drug or post-hoc analyses.

For experiment 1, groups of mice were injected with MA (30 mg/kg; 200 μl) or saline. One week post-injection mice were assessed for a working memory version of the RAM. Twenty-four hours before the second working memory assessment, mice were injected again with MA (30 mg/kg; 200 μl) or saline. For the remaining 3 weeks, mice were assessed weekly for working memory performance, as illustrated in the timeline (Figure 1A). We evaluate the % correct score for each trial, which is calculated as the number of total arm entries required to collect all 8 food rewards divided by the number of food rewards retrieved. We show the % correct scores in two separate analyses to illustrate the differences in number of errors committed while finding the first 4 food rewards (Figure 1B) when the working memory load is low, compared to the last 4 food rewards (Figure 1C) when the working memory load is high. The results shown in Figure 1C illustrate an overall significant effect of training [*F*_(7, 49)_ = 3.67, *n* = 4/group, *p* = 0.003], an overall significant improvement from MA [*F*_(1, 49)_ = 5.85, *n* = 4/group, *p* = 0.04] and a significant *post-hoc* effect at 1 week (Bonferroni corrected *t*-test = 3.23, *p* < 0.05). In collecting baits 1–4, mice from both treatment conditions perform equivalently (Figure 1B). Latency to complete the task shows an overall significant improvement over testing weeks [*F*_(7, 49)_ = 4.2, *n* = 4/group, *p* = 0.0001], no significant effects of drug treatment and no significant *post-hoc* comparisons (Figure 1D).

**Figure 2:**
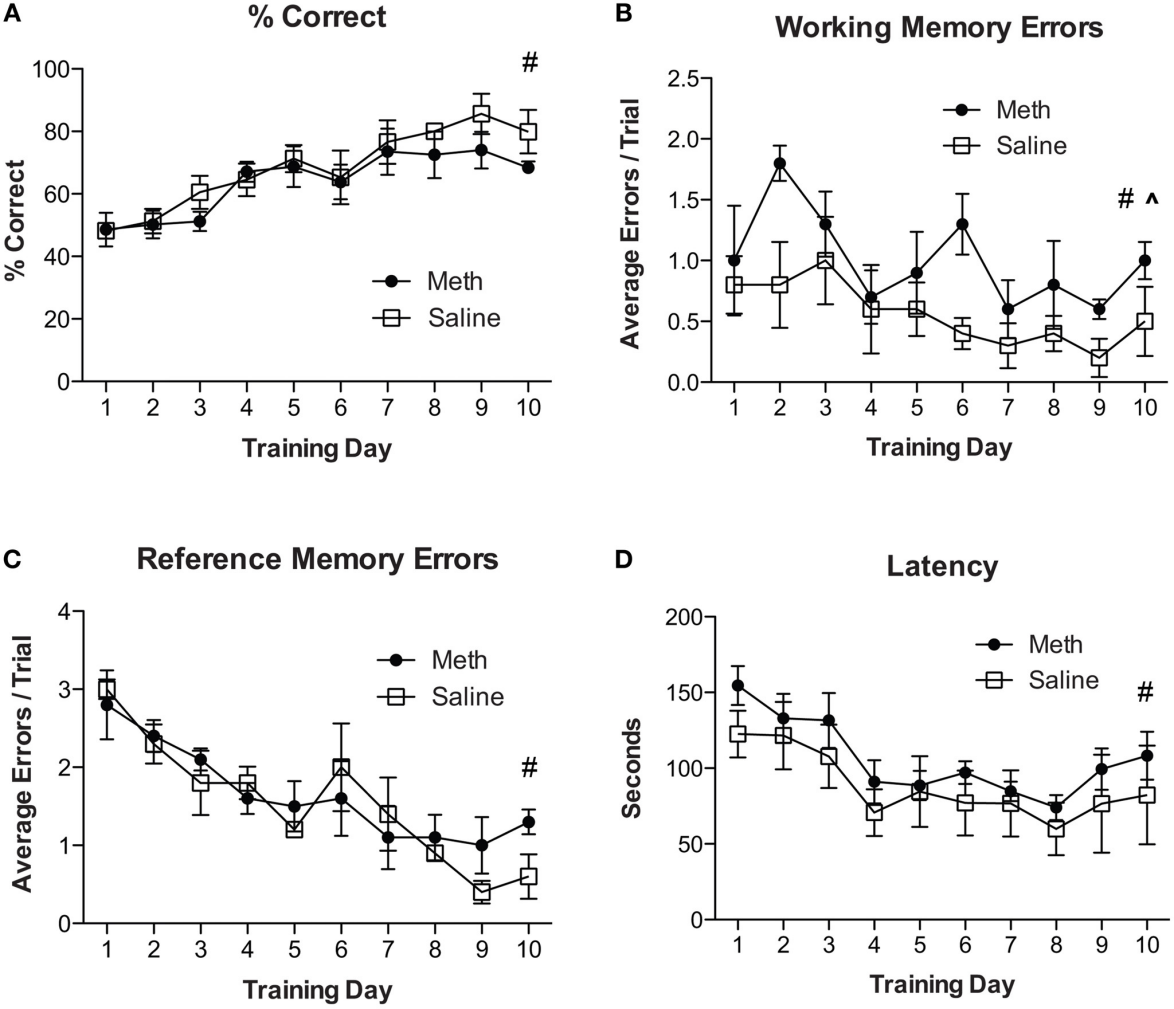
**MA increases working memory errors for cognitive flexibility task. (A)** Percent correct scores for both treatment conditions show an overall significant improvement over training days (^#^*p* < 0.01). There were no other significant effects. **(B)** Working memory errors show a significant overall reduction in errors over training days (**^#^***p* = 0.01) and a significant increase in working memory errors in MA treated mice compared to controls (^^^*p* < 0.05). **(C)** Reference memory errors show a significant overall effect of training (**^#^***p* < 0.01) and no other significant differences. **(D)** Latency to complete the trial shows an overall significant reduction in latency over training days (**^#^***p* < 0.01) and no other significant differences.

For experiment 2, all mice were re-trained on the RAM using a new configuration of four baited and four unbaited arms, which is different from having all arms baited as described in experiment 1. Mice were given 6 consecutive trials per day for 10 days. The results in Figure 2A show an overall significant improvement in % correct scores over training days [*F*_(9, 54)_ = 9.3, *n* = 4/group, *p* < 0.01]. There were no significant effects of drug treatment and no significant *post-hoc* analyses. Analyses for working memory errors (Figure 2B) show a significant overall reduction in errors over training days [*F*_(9, 54)_ = 3.0, *n* = 4/group, *p* = 0.01] and a significant increase in working memory errors in MA treated mice [*F*_(1, 54)_ = 6.0, *n* = 4/group, *p* < 0.05]. Analysis of reference memory errors (Figure 2C) show a significant overall reduction in errors over training days [*F*_(9, 54)_ = 12.92, *n* = 4/group, *p* < 0.01] and no significant drug treatment effects. Analyses of latency to complete the trial shows an overall significant reduction in latency over training days [*F*_(9, 54)_ = 14.05, *n* = 4/group, *p* < 0.01] and no other significant differences (Figure 2D).

**Figure 3:**
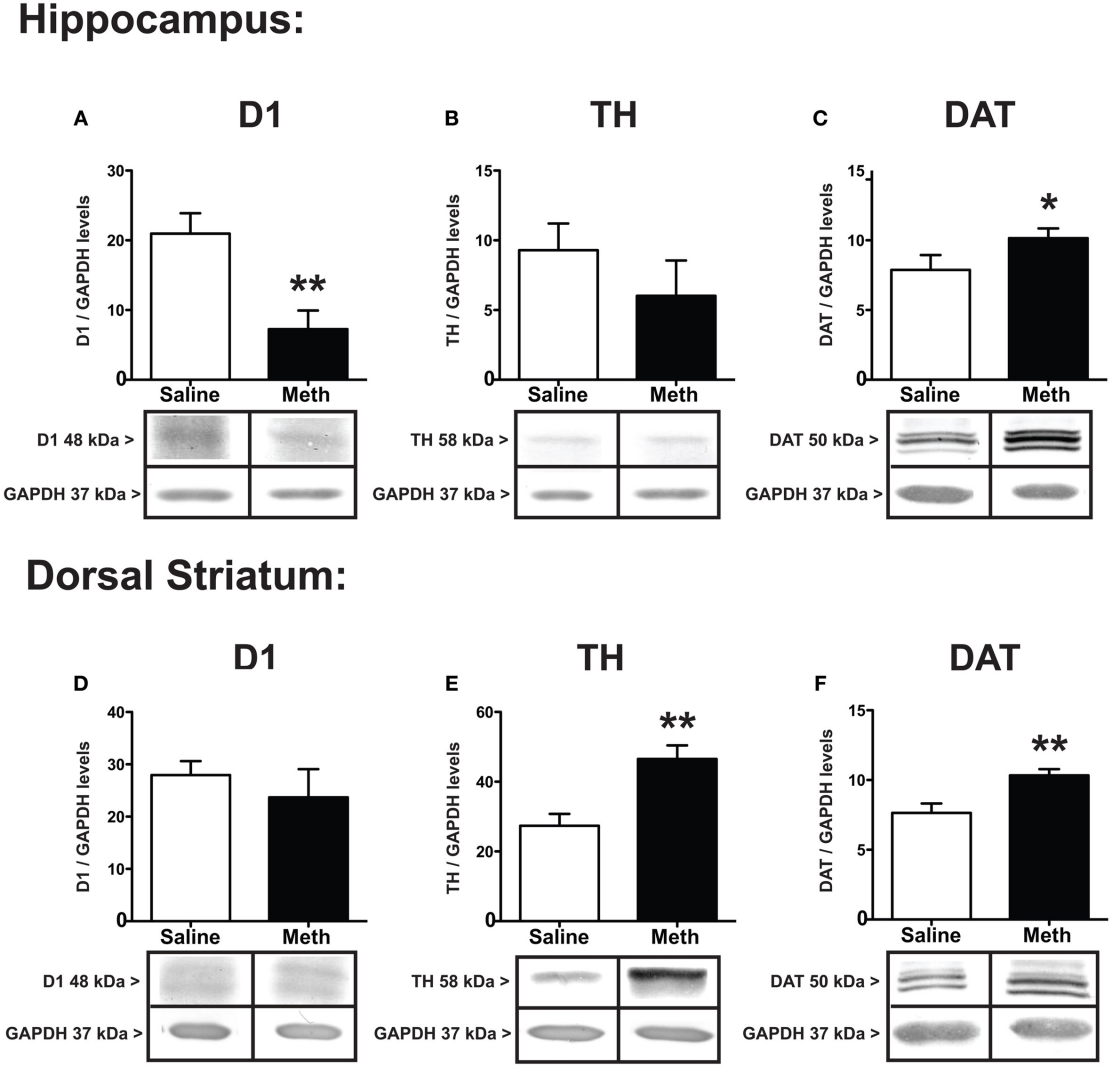
**Expression of dopamine markers**. D1, TH, and DAT in the hippocampus **(A–C)** and dorsal striatum **(D–F)** 7 weeks after the first injection of MA or saline. D1 in the hippocampus significantly decreased compared to controls (^**^p < 0.01), without changing expression in the dorsal striatum **(A,D)**. Compared to controls, TH levels significantly increased in the dorsal striatum (^**^p < 0.01) without changing expression levels in the hippocampus **(E,B)**. DAT increased significantly in both the hippocampus and dorsal striatum (**C,F**; ^*^p < 0.05; ^**^p < 0.01).

Immediately following the 60th RAM trial, all brains were microdissected for hippocampus and dorsal striatum. Figure 3 shows the protein expression differences between MA and saline treatments for D1, TH, and DAT. The results in the hippocampus show that D1 decreased in the hippocampus after MA exposure [*t*_(8)_ = 3.47, *p* < 0.01] without significant differences between groups in the dorsal striatum (Figures 3A,D). TH expression shows significant increases in the dorsal striatum after MA [*t*_(6)_ = 3.71, *p* < 0.001], without significant differences between treatment groups in the hippocampus (Figures 3B,E). Compared to saline controls the expression of DAT increased significantly after MA in both the hippocampus [*t*_(7)_ = 2.17, *p* < 0.05] and dorsal striatum [*t*_(6)_ = 3.31, *p* < 0.01] (Figures 3C,F).

**Figure 4:**
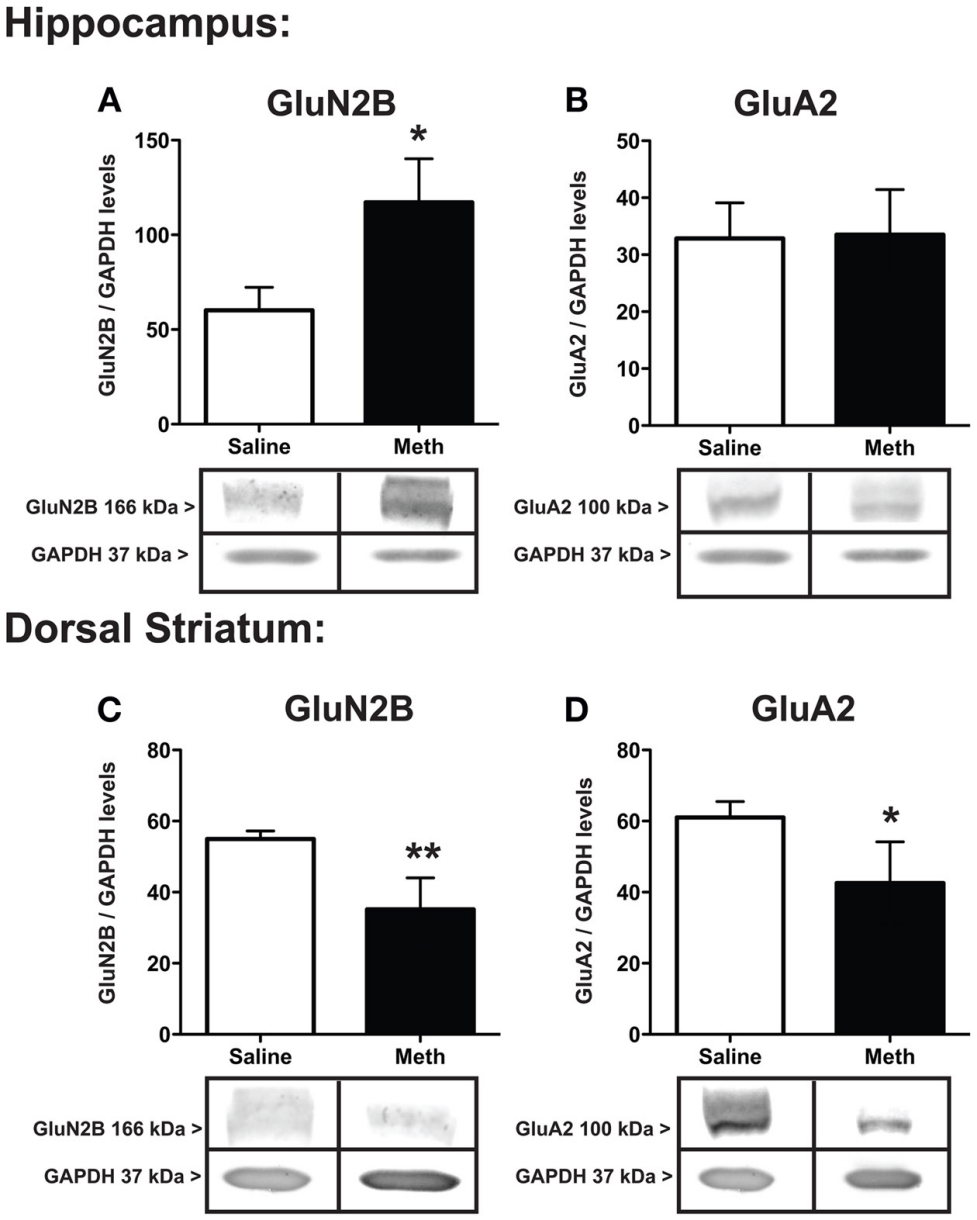
**Expression of glutamate receptor subunits**. GluN2B and GluA2 in the hippocampus **(A,B)** and dorsal striatum **(C,D)** 7 weeks after the first injection of MA or saline. The hippocampus shows significant increases in GluN2B with MA treatment (^*^p < 0.05) with a concomitant decrease in the dorsal striatum (^**^p < 0.01; **A,C**). GluA2 levels significantly decreased in the dorsal striatum after MA treatment (^*^p < 0.05) but did not significantly change between conditions in the hippocampus **(D,B)**.

The protein expression for the NMDA receptor subunit GluN2B, and the AMPA receptor subunit, GluA2, after saline or MA treatments are shown in Figure 4. The expression of GluN2B significantly increased within the hippocampus [*t*_(6)_ = 2.51, *p* < 0.05] and significantly decreased within the dorsal striatum [*t*_(6)_ = 3.66, *p* < 0.01] after MA treatment compared to controls. In the hippocampus GluA2 expression was not significantly different between conditions, while in the dorsal striatum MA treatment significantly decreased GluA2 [*t*_(6)_ = 2.08, *p* < 0.05].

**Figure 5:**
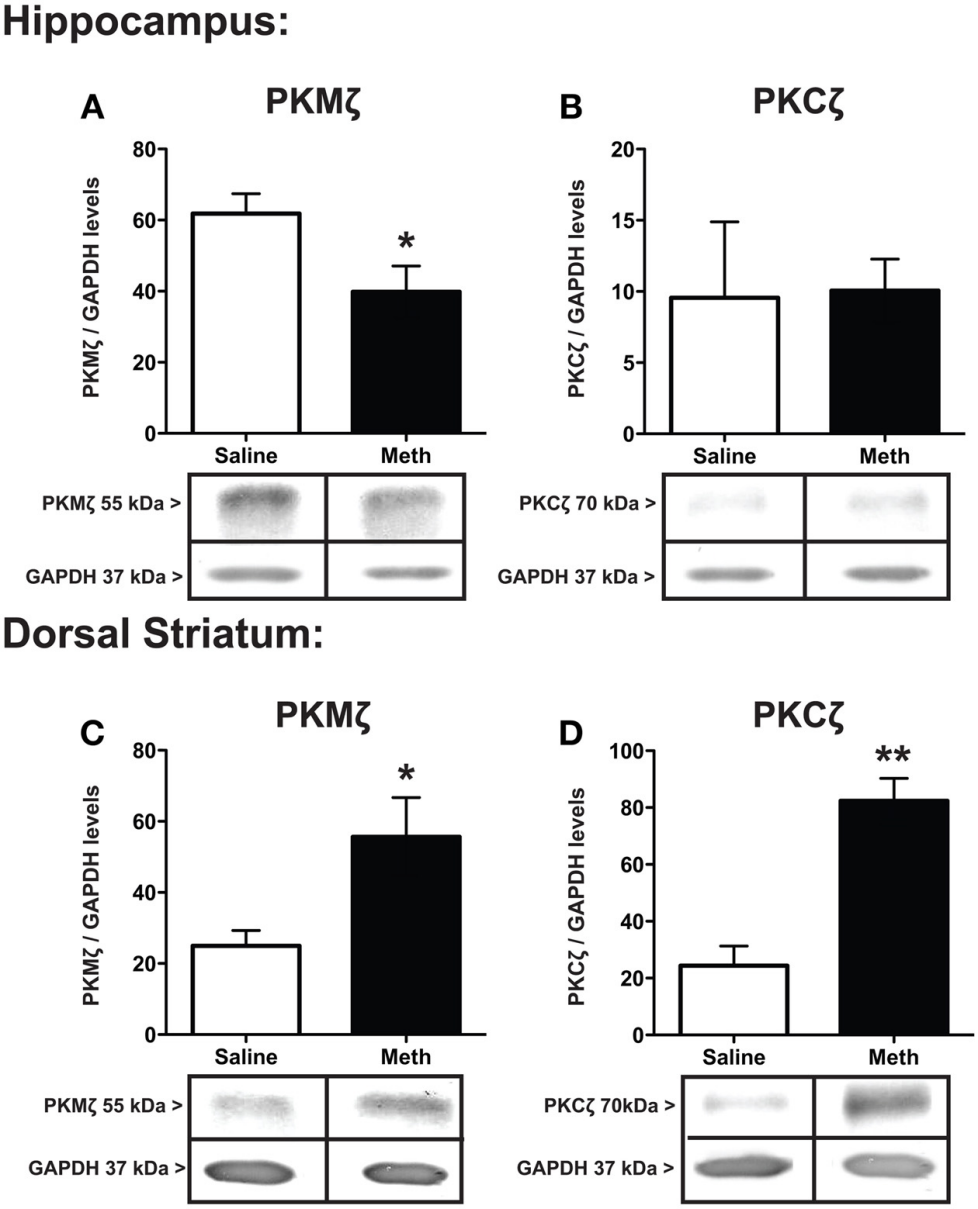
**Expression of PKMζ/PKC ζ**. PKMζ and PKCζ in the hippocampus **(A,B)** and dorsal striatum **(C,D)** 7 weeks after the first injection of MA or saline. The hippocampus shows significant decrease in PKMζ with MA treatment (^*^p < 0.05) with a concomitant increase in the dorsal striatum compared to controls (^*^p < 0.05; **A,C**). PKCζ levels significantly increased in the dorsal striatum after MA treatment (^**^p < 0.01) but did not significantly change between conditions in the hippocampus **(D,B)**.

Protein expression for PKMζ and PKCζ within the hippocampus and dorsal striatum after saline or MA treatments is shown in Figure 5. The results show a significant decrease in hippocampal PKMζ [*t*_(6)_ = 2.39, *p* < 0.05] expression with a concomitant increase in the dorsal striatum compared to control treatment [*t*_(6)_ = 2.58, *p* < 0.05] (Figures 5A,C). PKCζ did not change significantly between conditions in the hippocampus (Figure 5B) but significantly increased after MA treatment in the dorsal striatum [*t*_(6)_ = 5.53, *p* < 0.01].

## Discussion

### Memory enhancing effects of MA

Behaviorally we show that MA improves working memory performance 1 week after the first MA bolus injection (30 mg/kg). This effect is consistent with clinical studies identifying short-term cognitive enhancing effects for learning and memory, visuospatial perception, and response speed after limited and low-dose stimulant exposure (Johnson et al., 2000; Silber et al., 2006; Mahoney et al., 2010; Marrone et al., 2010; Hart et al., 2011; Kirkpatrick et al., 2011). Conversely, chronic MA users have cognitive deficits in sustained attention, episodic memory, information processing, and impulse control (Nordahl et al., 2003; Monterosso et al., 2005; Simon et al., 2010; Morgan et al., 2012). Many of these results are also shown in rodent studies (Mahoney et al., 2010; Hart et al., 2011). We also show that 7 weeks after MA exposure there are significant cognitive deficits on the reference and working memory version of the 8-arm radial maze. In this assessment the RAM had only 4 baited arms, which is different from the initial configuration of the maze where all arms were baited. This required mice to relearn the RAM during the 10 consecutive days of training with 6 daily trials, which tests cognitive flexibility. The behavioral results show that MA-treated mice produce significantly more working memory errors during training days. This is consistent with the behavioral effects of MA that have been reported in both humans (Meredith et al., 2005) and other animals (Simões et al., 2007; González et al., 2014), and particularly in MA-treated animals learning a cognitive task with changes in reward contingencies (Stolyarova et al., 2014) and reversal learning (Kosheleff et al., 2012).

### Specificity of dopamine toxicity by MA

Rapid effects of neurotoxic dosages of MA are associated with decreases in DA terminals (O'Callaghan and Miller, 1994; Sonsalla et al., 1996; Fumagalli et al., 1998; Wallace et al., 1999), the DA precursor, TH, and the reuptake transporter mechanism, DAT (Cadet and Krasnova, 2009). Because of these rapid effects of MA it is expected that there may be compensatory mechanisms that would change the levels of either the presynaptic mechanisms involved in dopamine release and/or in postsynaptic dopamine receptor dynamics. We find that several weeks after MA treatment, both the hippocampus and dorsal striatum show effects of compensation involving DAT, TH, and D1 expression. It is important to note that there were no deaths or seizures associated with either MA injections and no fever was mounted by any of these animals. The lack of these behavioral indices after MA exposure is associated with producing lower levels of Fluoro-Jade positive cells in rats that did not show evidence of blood-brain barrier disruption concomitant with hyperthermia and seizures (Bowyer and Ali, 2006). However, due to the longer time points we examined it would presumably allow deficits to develop over time.

### Effects of MA-induced dopamine reduction in hippocampus and dopamine increase in striatum

Within the hippocampus, the D1 receptor is downregulated compared to controls with a concomitant increase in cytosolic DAT expression. This could reflect enhanced endocytosis or faster DAT kinetics resulting in lower membrane expression and dampening the signaling of dopamine consistent with other reports (Silva et al., 2014). Faster DAT kinetics could also mediate the downregulation of D1 by increasing turnover and uptake of dopamine by the transporter. It is known that excessive levels of dopamine or moderate levels can impair cognitive performance (Arnsten, 1998). Moreover, downregulation of D1 significantly impairs spatial learning (Furini et al., 2014). D1-deficient mice show impairment in associative learning and synaptic plasticity in the CA3-CA1 synapses (Ortiz et al., 2010), and impairments in CA1 long term potentiation (LTP; Ghanbarian and Motamedi, 2013). Additionally, downregulation of DAT disrupts spatial learning and retention (Del'Guidice et al., 2013) as well as showing deficits in cognitive flexibility (Morice et al., 2007). We speculate that the downregulation of D1 and the upregulation of DAT endocytosis occur as a consequence of MA and is a contributing factor in spatial working memory deficits.

In the dorsal striatum there were no changes in D1 expression compared to controls, rather, there was a significant increase in TH levels and DAT endocytosis. This suggests that in the striatum, MA is upregulating presynaptic mechanisms involving the synthesis and degradation of dopamine. These presynaptic changes are potential compensatory mechanisms to the rapid neurotoxic effects of MA. While MA is known to damage DA terminals without affecting postsynaptic receptors (Cadet et al., 2003; Krasnova and Cadet, 2009; Sulzer, 2011), many of these DA terminals partially recover after MA (Ares-Santos et al., 2014). The significant increase in TH and the increased endocytosis of DAT suggests that MA induces DAT hyperfunction in the striatum. DAT hyperfunction has been associated with a model of attention deficit hyperactivity disorder (ADHD) in rats that also show a working memory deficit (Ruocco et al., 2014). Together these data indicate that DAT, TH, and D1 dysregulation within the hippocampus and dorsal striatum could collectively play a role in the working memory deficit observed after weeks of MA abstinence.

### Effects of MA-induced GluN2B increase in hippocampus and decrease of GluN2B and GluA2 in striatum

Our results show that MA treatment significantly increased GluN2B subunit expression in the hippocampus with a concomitant decrease in the striatum. These data are consistent with reports showing differential effects on glutamatergic excitotoxicity between the hippocampus and striatum (Yamamoto et al., 1999). Changes in NMDA receptor expression is expected since these receptor subunits can regulate excitotoxic effects (Lynch and Guttmann, 2002; Silva, 2003) and are known to change in expression after MA exposure (Bowers et al., 2010; Kalivas and Volkow, 2011). The GluN2B subunits modulate the electrophysiological properties of the NMDA channels involving Ca^2+^ permeability (Dingledine et al., 1999). This receptor subunit forms heterodimers with GluN1 and GluN2B and is important for long-term depression (Liu et al., 2004). However, other reports show that the GluN2B subunit is critical for spatial learning and LTP (Clayton et al., 2002). Overexpresison of GluN2B improved spatial learning and enhanced LTP (Tang et al., 1999), and working memory (Wang et al., 2008, 2013). This suggests that MA may be interrupting the hippocampal plasticity by increasing the Ca^2+^ influx through GluN2B NMDA receptor, leading to excitotoxicity and negatively affecting working memory performance (Nabekura et al., 2002). In our study, MA treatment occurred during juvenile development. During juvenile development the availability of GluN2B is particularly important in the prefrontal cortex in the expression of LTP (Flores-Barrera et al., 2013). These reports suggest that the decrease in GluN2B that we observe within the striatum may be compromising the developmental switch from juvenile NMDA function to that of an adult that involves longer lasting NMDA responses and increased GluN2B subunit expression (Flores-Barrera et al., 2013). While our tissues were from the dorsal striatum and not specifically frontal cortex, these reports may still be relevant to our findings.

Our results also identify a significant decrease in AMPA GluA2 subunits within the striatum without significant changes in the hippocampus. Reports show that MA exposure involving escalating doses for 1 week increases GluA2 protein expression (Simões et al., 2008), while MA exposure following 2 weeks of escalating doses decreases GluA2 involving epigenetic factors (Jayanthi et al., 2013). The latter report is consistent with the effects we show here. These reports suggest that our acute MA exposure may produce excitotoxic damage that continues to develop over several weeks that result in similar effects on AMPA receptor changes that are associated with 2 weeks of MA exposure (Jayanthi et al., 2013). It remains to be seen whether the same epigenetic factors are involved in the downregulation of the GluA2 subunit with acute MA exposure followed by long-term abstinence as we model here.

### Effects of MA-induced dysregulation of PKMζ

We find that MA has significant effects on cognitive flexibility involving PKMζ, an atypical kinase that is important for long-term memory maintenance (Pastalkova et al., 2006; Shema et al., 2007; Serrano et al., 2008; Sebastian et al., 2013a,b). The significant decrease in PKMζ within the hippocampus of MA mice compared to controls suggests that MA may be inhibiting PKMζ directly or indirectly during training. It has been shown that the expression of PKMζ within the hippocampus is correlated with memory performance on the RAM (Sebastian et al., 2013a). This is consistent with our results showing reduced PKMζ in MA-treated mice that also show increased working memory errors. In the hippocampus, PKCζ did not change between conditions. The effects of MA in the striatum upregulate both PKMζ and PKCζ which we find may be coupled to both D1 and GluN2B expression.

### The role of PKMζ/PKCζ in the D1/GluN2B complex

D1 receptors and NMDA receptors co-immunoprecipitate (Fiorentini et al., 2003) and are co-localized in several brain structures, including the striatum and hippocampus (Gracy and Pickel, 1996; Cepeda and Levine, 1998; Sesack et al., 2003). The D1 receptor stimulates protein kinase A (PKA) and enhances NMDA GluN2B currents via protein kinase C (PKC)-dependent mechanisms (Chen et al., 2004; Liu et al., 2007). Moreover, this effect is reversed by chelerythrine (Gu et al., 2007) at a dose that selectively inhibits PKMζ (Serrano et al., 2005). This suggests that PKMζ/PKCζ may be involved in the phosphorylation of D1/GluN2B complexes. In the striatum we show a decrease in GluN2B with MA treatment suggesting that the ratio between available GluN2B and D1 receptors is off balance resulting in reduced receptor function (Gu et al., 2007) and potentially contributing to the deficits in learning we report. Additionally, we find that within the dorsal striatum, levels of PKMζ and PKCζ are both significantly elevated with MA treatment. It is known that increased levels of these kinases can decrease DAT function (Daniels and Amara, 1999; Melikian and Buckley, 1999) by accelerating internalization (Holton et al., 2005; Sorkina et al., 2005), reducing recycling (Loder and Melikian, 2003), and/or increasing degradation (Miranda et al., 2005) which could create another source of dysregulated dopamine function contributing to the behavioral changes we identify.

## Conclusion and clinical implications

Acute MA administration induced a cognitive enhancing effect on working memory performance at 1 week post MA administration. Over the subsequent weeks, this memory enhancing effect diminished and a working memory deficit manifested during a cognitive flexibility test. The protein analysis of tissues from both the hippocampus and striatum show divergent effects of MA treatment on all receptors tested: D1, GluN2B subunit and GluA2 subunit, and divergent effects with PKMζ, PKCζ, and TH. Only the DAT cytosolic expression was consistent between both brain regions. These data identify that short-term acute bolus dose of MA followed by long-term abstinence can continue to manifest deficits in both dopaminergic and glutamatergic signaling involving PKMζ and PKCζ. Dysregulating dopaminergic signaling with MA could contribute to dopamine-related pathologies. This is consistent with the findings that MA addicts with low levels of dopamine have higher incidents of depression (Zhang et al., 2014) and cognitive deficits (Obermeit et al., 2013), both of which are comorbid (Casaletto et al., 2014). These lower levels of DA signaling also create significant risk factors for developing Parkinson's disease (Callaghan et al., 2012).

## Author contributions

Damian Drapala, Dr. Ingrid Tulloch, and Dr. Peter Serrano designed the experiments. Damian Drapala conducted all the behavioral testing. Dr. Ingrid Tulloch performed all the microdisections. Stephen Braren and Dr. Peter Serrano fractionated all samples, performed western blots, analyzed the results and wrote the manuscript. All authors approved the final manuscript for submission.

### Conflict of interest statement

The authors declare that the research was conducted in the absence of any commercial or financial relationships that could be construed as a potential conflict of interest.
