# Supplementary material for: Methamphetamine-induced short-term increase and long-term decrease in spatial working memory affects protein Kinase M zeta (PKMζ), dopamine, and glutamate receptors
Source: Front Behav Neurosci. 2014 Dec 18;8:438. doi: 10.3389/fnbeh.2014.00438 (PMC4270177; doi:10.3389/fnbeh.2014.00438)
Supplement: Supplementary file 1 [file Image1.PDF]

Supplementary Material

Methamphetamine-induced short-term increase and long-term decrease in spatial working memory affects Protein Kinase M zeta (PKMζ), dopamine, and glutamate receptors

Stephen H. Braren [1], Damian Drapala [1], Ingrid K. Tulloch[3] and Peter A. Serrano [1,2*]

[1]Department of Psychology, Hunter College, New York, NY
[2]The Graduate Center of CUNY, New York, NY
[3]Department of Psychology, Stevenson University, Baltimore, MD

*Correspondence: Peter A. Serrano, Hunter College, Department of Psychology, 695 Park Avenue, New York, NY 10065, USA.
serrano@genectr.hunter.cuny.edu

1. Supplementary Figure

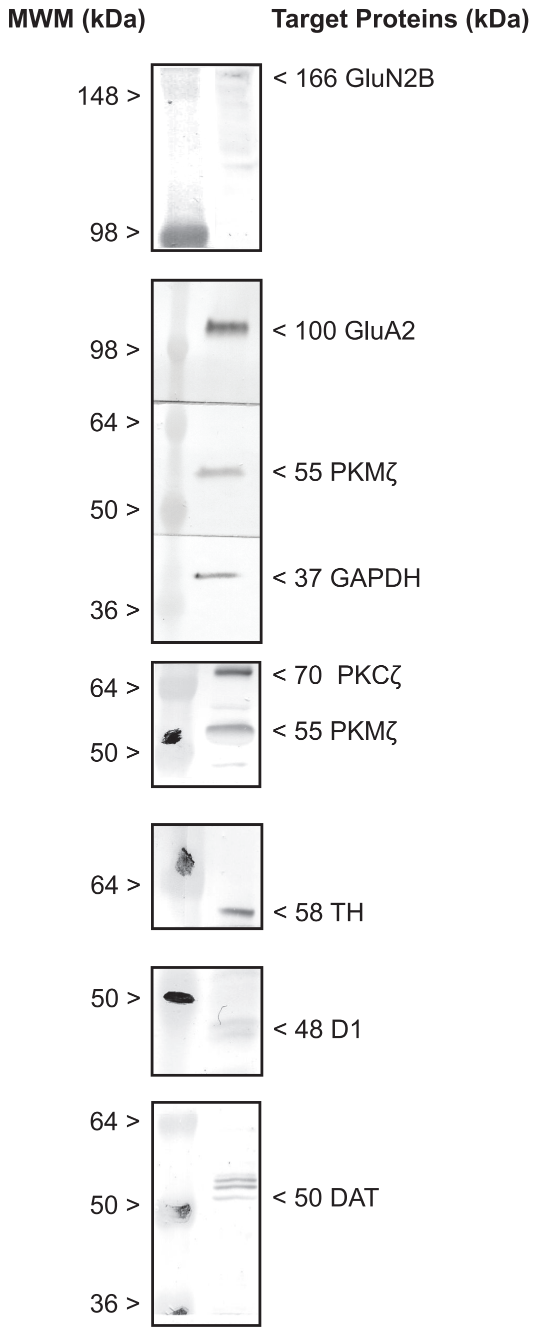

Supplementary Figure 1: Protein molecular weight marker (MWM; Seeblue plus 2, Life Technologies, Carlsbad, CA) used to identify various target proteins on representative immunoblots.
